# Supplementary material for: Immigration rates and species niche characteristics affect the relationship between species richness and habitat heterogeneity in modeled meta-communities
Source: PeerJ. 2015 Mar 10;3:e832. doi: 10.7717/peerj.832 (PMC4359120; doi:10.7717/peerj.832)
Supplement: Figure S1 [file peerj-03-832-s001.pdf]

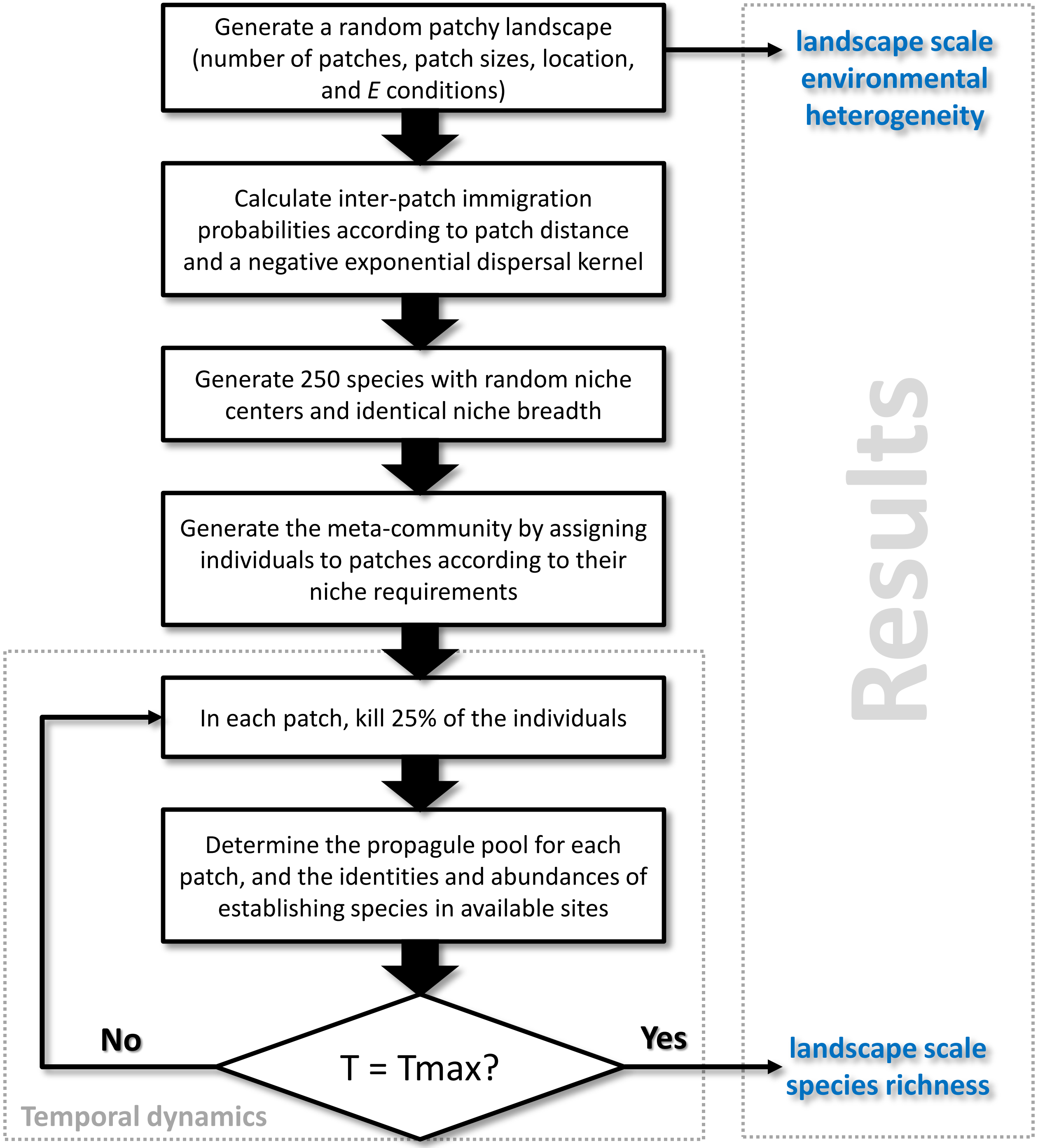
Generate a random patchy landscape (number of patches, patch sizes, location, and E conditions)
landscape scale environmental heterogeneity
Calculate inter-patch immigration probabilities according to patch distance and a negative exponential dispersal kernel
Generate 250 species with random niche centers and identical niche breadth
Generate the meta-community by assigning individuals to patches according to their niche requirements
In each patch, kill 25% of the individuals
Determine the propagule pool for each patch, and the identities and abundances of establishing species in available sites
T = Tmax?
No
Yes
landscape scale species richness
Temporal dynamics
Results
