# Supplementary figures and images for: Immigration rates and species niche characteristics affect the relationship between species richness and habitat heterogeneity in modeled meta-communities
Source: PeerJ. 2015 Mar 10;3:e832. doi: 10.7717/peerj.832 (PMC4359120; doi:10.7717/peerj.832)

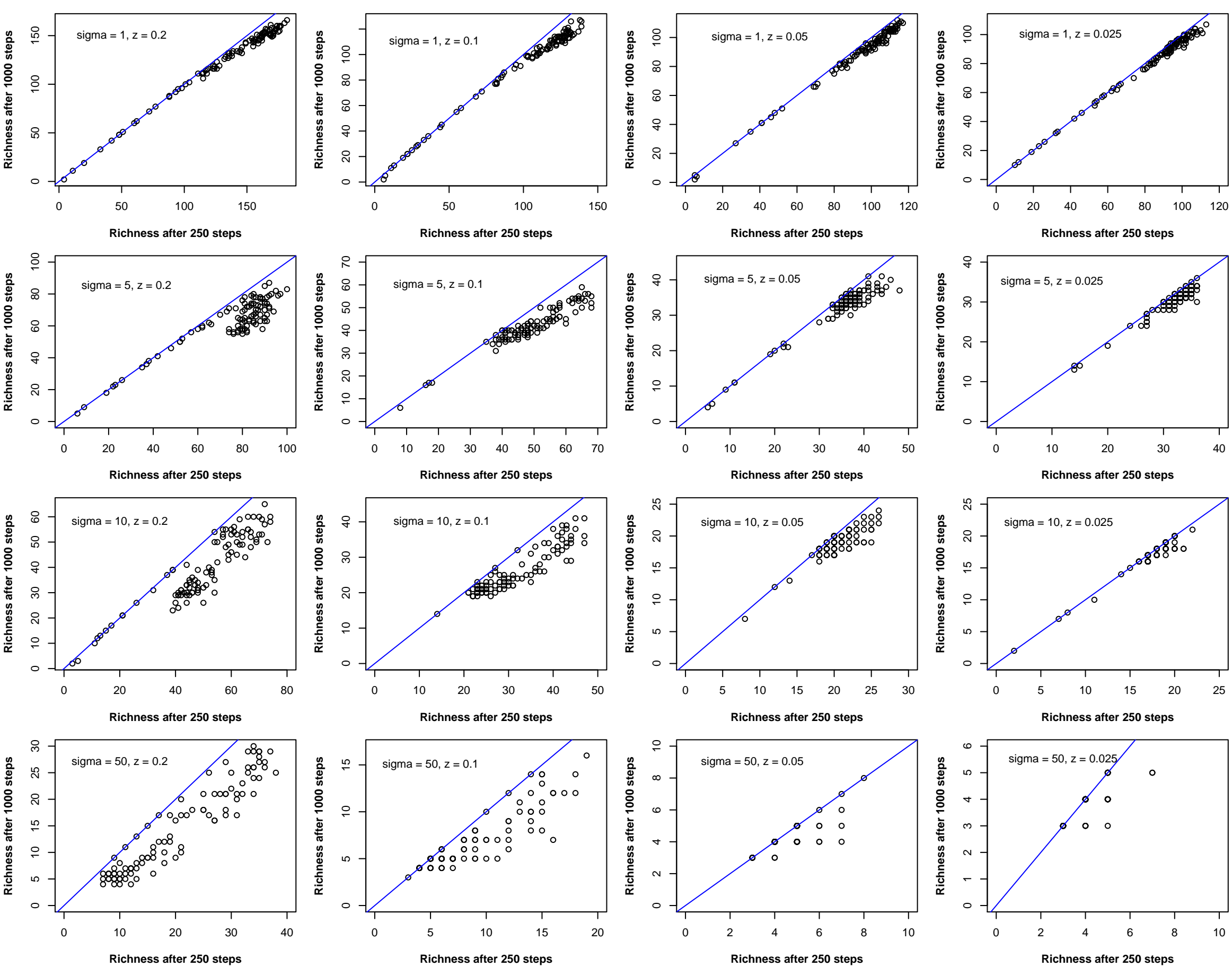

Supplement: Figure S2 — Species richness levels after 250 and 1,000 time steps in model simulations with different combinations of species niche breadth and immigration rates. Each circle is based on the same landscape, i.e., it represents results with identical levels of heterogeneity, but different simulation lengths. The blue lines depict the 1:1 relationship. [file peerj-03-832-s002.pdf]
