# Supplementary material for: Immigration rates and species niche characteristics affect the relationship between species richness and habitat heterogeneity in modeled meta-communities
Source: PeerJ. 2015 Mar 10;3:e832. doi: 10.7717/peerj.832 (PMC4359120; doi:10.7717/peerj.832)
Supplement: Figure S3 — The figure is based on simulations with 250 time steps (compare with Fig. 2 in the main text). (A–E) correspond with different species niche widths (A, E—very narrow, B, F—narrow, C, G—intermediate, and D, H—wide). Curves denote inter-patch immigration rates, with circle colors depicting the value of the z parameter (0.2—black, 0.1—blue, 0.05—green, and 0.025—white, reflecting increasing levels of inter-patch immigration rates). The top row is based on Shannon’s measure of heterogeneity, while the bottom row uses patch richness as the heterogeneity measure. [file peerj-03-832-s003.pdf]

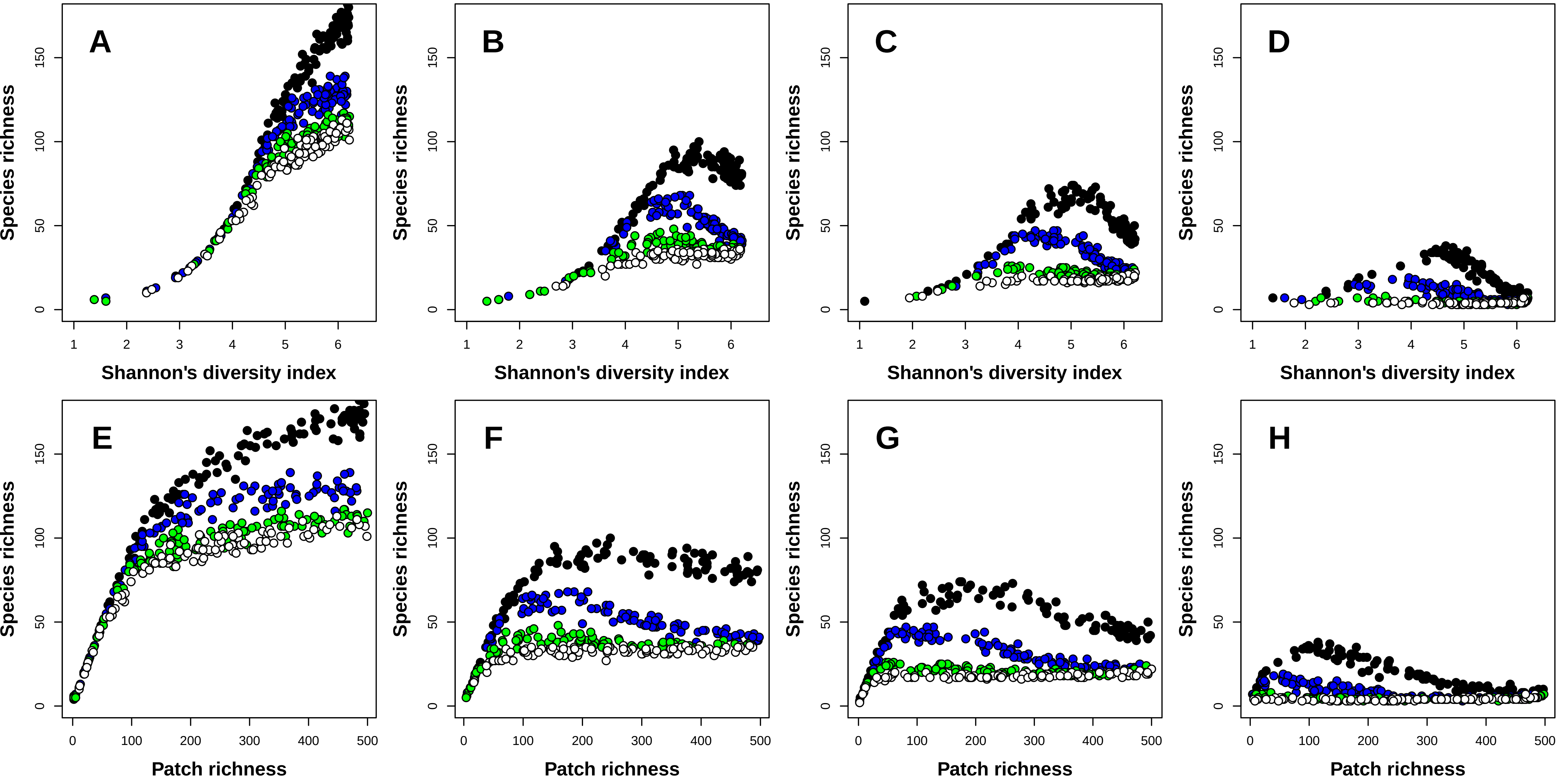
A
B
C
D
E
F
G
H
Species richness
Shannon's diversity index
Patch richness
0
50
100
150
1
2
3
4
5
6
0
100
200
300
400
500
